# Non-invasive brain stimulation and computational models in post-stroke aphasic patients: single session of transcranial magnetic stimulation and transcranial direct current stimulation. A randomized clinical trial

**DOI:** 10.1590/1516-3180.2016.0194060617

**Published:** 2017-11-06

**Authors:** Michele Devido dos Santos, Vitor Breseghello Cavenaghi, Ana Paula Machado Goyano Mac-Kay, Vitor Serafim, Alexandre Venturi, Dennis Quangvinh Truong, Yu Huang, Paulo Sérgio Boggio, Felipe Fregni, Marcel Simis, Marom Bikson, Rubens José Gagliardi

**Affiliations:** I PhD. Professor of Speech Pathology and Audiology, Faculdade de Ciências Médicas da Santa Casa de São Paulo (FCMSCSP), São Paulo (SP), Brazil.; II Medical Student, Faculdade de Ciências Médicas da Santa Casa de São Paulo (FCMSCSP), São Paulo (SP), Brazil.; III PhD. Professor of Speech Pathology and Audiology, Universidad Santo Tomás, Viña del Mar, Chile.; IV PhD. Biomedical Engineer, Engineering Department, City College of New York, New York, United States.; V BSc, PhD. Professor of Cognitive Neuroscience, Cognitive Neuroscience Laboratory, Mackenzie Presbyterian University, São Paulo (SP), Brazil.; VI MD, PhD, MPH. Associate Professor of Physical Medicine & Rehabilitation, Associate Professor of Neurology, Harvard Medical School; Director Neuromodulation Center, Spaulding Rehabilitation Hospital, Harvard Medical School, Boston, Massachusetts, United States.; VII MD, PhD. Neurologist, Irmandade da Santa Casa de Misericórdia de São Paulo, and Instituto de Medicina Física e Reabilitação (IMREA), Hospital das Clínicas (HC), Faculdade de Medicina da Universidade de São Paulo (FMUSP), São Paulo (SP), Brazil.; VIII PhD. Associate Professor of Biomedical Engineering, City College, City University of New York, New York, United States.; IX MD, PhD. Full Professor, Department of Neurology, Faculdade de Ciências Médicas da Santa Casa de São Paulo (FCMSCSP), São Paulo (SP), Brazil.

**Keywords:** Aphasia, Stroke, Speech disorders, Transcranial direct current stimulation, Transcranial magnetic stimulation

## Abstract

**CONTEXT AND OBJECTIVE:**

Patients undergoing the same neuromodulation protocol may present different responses. Computational models may help in understanding such differences. The aims of this study were, firstly, to compare the performance of aphasic patients in naming tasks before and after one session of transcranial direct current stimulation (tDCS), transcranial magnetic stimulation (TMS) and sham, and analyze the results between these neuromodulation techniques; and secondly, through computational model on the cortex and surrounding tissues, to assess current flow distribution and responses among patients who received tDCS and presented different levels of results from naming tasks.

**DESIGN AND SETTING:**

Prospective, descriptive, qualitative and quantitative, double blind, randomized and placebo-controlled study conducted at Faculdade de Ciências Médicas da Santa Casa de São Paulo.

**METHODS:**

Patients with aphasia received one session of tDCS, TMS or sham stimulation. The time taken to name pictures and the response time were evaluated before and after neuromodulation. Selected patients from the first intervention underwent a computational model stimulation procedure that simulated tDCS.

**RESULTS:**

The results did not indicate any statistically significant differences from before to after the stimulation. The computational models showed different current flow distributions.

**CONCLUSIONS:**

The present study did not show any statistically significant difference between tDCS, TMS and sham stimulation regarding naming tasks. The patients’ responses to the computational model showed different patterns of current distribution.

## INTRODUCTION

Transcranial magnetic stimulation (TMS) and transcranial direct current stimulation (tDCS) are safe non-invasive techniques that present different characteristics. TMS equipment is more expensive, its stimulation is more focal, it has better temporal resolution and its accuracy is of the order of milliseconds while that of tDCS is of the order of minutes. TMS generates muscle contraction and provides a sound stimulus during its application, while TDCS is more easily ­applicable, does not generate muscle contraction and does not provide back-sound stimulus. In applications of TMS, rare cases of convulsion have been reported although mild adverse effects (such as transient headache) may occur. The literature on tDCS does not report any correlation with seizure but it describes mild adverse effects such as transient headache. Both techniques can influence distant cortical and subcortical areas beyond the stimulation area, due to trans-synaptic effects. The current direction may differ in subjects with or without brain injury.^1^^,^^2^

The concept of inter-hemispheric competition for language and motor deficits after stroke lies behind the principle of neuromodulation. The aim is to facilitate increased brain activity in the injured hemisphere, while favoring inhibition of cortical activity in the healthy hemisphere.^3^^,^^4^^,^^5^

Investigations using one session of TMS or tDCS have suggested that use of these techniques among aphasic patients after stroke has a relationship with language improvement. Thus, these techniques may be promising for speech rehabilitation in cases of aphasic syndromes.^6^^,^^7^ Recent evidence from noninvasive brain stimulation (NIBS) has indicated that neuromodulation in consecutive sessions might be a beneficial tool for improving language skills among aphasic patients.^8^^,^^9^

Inter-individual differences in response to NIBS remain an important area of investigation and a hurdle to be surmounted for achieving clinically efficacious treatment. The reasons for different responses shown in studies are not well-defined, but may relate to the distribution of electric current through the brain and surrounding tissues. Thus, development of computational models simulating the current distribution of tDCS in patients with different clinical responses makes it possible to accurately review current patterns in tDCS applications and to understand clinical outcomes better.^10^

## OBJECTIVES

The aim of this study was firstly to describe the responses of significantly aphasic post-stroke patients to naming tasks, before and after one application of tDCS, TMS or sham, and to compare these neuromodulation techniques; secondly, the intention was to describe current distribution in the cortex and surrounding tissues through computational model stimulation, among patients who showed different results in naming tasks after tDCS.

## METHODS

This was a prospective, qualitative and quantitative, double blind, randomized and placebo-controlled study. It was approved by the research ethics committee of Santa Casa de São Paulo under registration number 169/10. Patients were randomized to receive the three forms of stimulation: tDCS, TMS and sham. The randomization was made by statistic orientation in three weeks (Table 1).

**Table 1.**
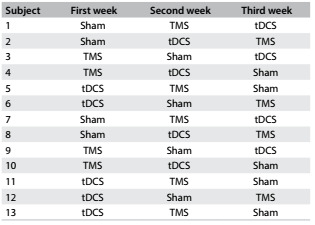
Description of the randomization

TMS = transcranial magnetic stimulation; tDCS = transcranial direct current stimulation.

The sample included post-stroke patients of both sexes who had suffered a left hemisphere ischemic stroke at least six months earlier. These patients were recruited in the Neurology Department and the sample size was calculated statistically. The diagnosis of aphasia was made by a speech pathologist before neuromodulation, and a medical neuroimaging evaluation was also performed. The lesions were located not only in the frontal lobe but also in the parietal lobe, temporal lobe and subcortical areas.

For the purposes of the present study, clinical and diagnostic findings of aphasia were given preference, rather than topographic data. The inclusion criteria were that the subjects needed to present Broca or anomic aphasia without comorbidities such as dysarthria or apraxia of speech and without previous speech and language therapy. Patients with any clinically significant or unstable medical or psychiatric disorder, any history of substance abuse or any neuropsychiatric comorbidity were excluded. The aphasia classification was based on speech and language pathology standards.^11^^,^^12^^,^^13^

Direct current stimulation was transferred through a saline-soaked pair of surface sponge electrodes (10 cm x 10 cm and 5 cm x 7 cm) and was delivered by means of a specially developed direct current stimulator. The electrode placement was as follows: the anode (10 cm x 10 cm) was over the Broca area and the cathode (5 x 7 cm) was centered horizontally over the F8 of the 10-20 system.^14^ tDCS was applied for 20 minutes at a current intensity of 2 mA. TMS was carried out in the right hemisphere, in the area homologous to Broca’s area, located by means of the 10-20 system (F8), with a frequency of 1 Hz, using 90% of the motor threshold for 20 minutes. The threshold corresponded to a lower-intensity stimulus applied to the right hemisphere motor area, which causes contraction of the left-side first interosseous muscle, observed through electromyography (EMG), using surface electrodes. The motor threshold was recalculated after test assessments. Motor cortex excitability was measured by means of the motor evoked potential (MEP) and silent period (SP) before starting and immediately at the end of stimulation.^15^

The tDCS placebo consisted of the same stimulator apparatus as described above, with the stimulator turned on for 20 seconds to mimic the effect of stimulation. The TMS placebo comprised a specific coil, with a screen that did not allow passage of the magnetic field but produced a sound stimulus of similar characteristics.

The subjects did the Boston Naming Test^16^ before and after each neuromodulation procedure*.* The patients received tDCS, TMS or placebo in a silent and well-lit room. Their responses were recorded with a head microphone in the CronoFonos software.

An exploratory analysis verified the scores before and after each picture-naming stimulation, considering not only the picture naming but also the picture-naming strategy (i.e. the number of words correctly named plus the linguistic strategies used by the subject to achieve this) and the response time (including the response time for naming strategy and total response time). In the event of absence of responses, the time interval was replaced by 20 seconds for each item unanswered.

The Kruskal-Wallis and Wilcoxon tests were used to compare responses and variables. The data were reported as means and standard deviations. Statistical significance was taken to be a two-tailed P-value of < 0.05.

Three subjects from the first intervention were selected to receive computational models that simulated the tDCS brain current flow. The criterion for selecting them was that they should be one of the best responders, the worst responder and a control, after one session of tDCS. The response classification considered the qualitative descriptive improvement/worsening of all parameters evaluated before and after the stimulation.

To calculate the tDCS-induced electric fields, a 3D model for the volume conductor (resolution of 1 mm^3^) was chosen. The entire process followed a previous study.^17^ The electrical properties of the tissues were representative of isotropic average values (in S/m): brain: 0.2; cerebrospinal fluid (CSF): 1.65; skull: 0.01; and scalp: 0.465. The muscle, fatty tissue, eye and blood vessel compartments paralleled the same scalp tissue. The anode (10 cm x 10 cm) was placed over the Broca area and the cathode (5 cm x 7 cm) was centered horizontally over the F8 of the 10-20 system. To implement the model, the commercially available Comsol Multiphysics 3.5 finite element (FE) package (Comsol Inc., MA, USA) was used, following a method that had already been described^18^. The results were compared on the same scale after the simulation and the current density ranged from 0 V/m to 0.522 V/m.

## RESULTS

Thirteen patients were included in this study (53.8% men), with a mean age of 56 years and with elementary and high school educational levels. All the patients received active transcranial direct current stimulation, transcranial magnetic stimulation or sham, with no adverse effects reported. Table 2 describes the subject data and Table 1 shows the randomization results.

**Table 2.**
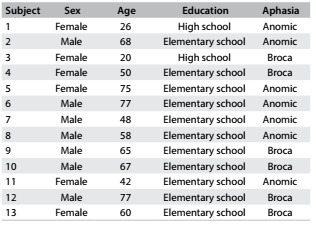
Description of the patients

Table 3 details the statistical results from the Wilcoxon test on mean performance in the naming test before and after stimulation. These assessments indicated that there was a statistically significant difference in the picture-naming task after a single application of tDCS, and this was also seen from the sham. Comparison of the three techniques using the Kruskal-Wallis test did not demonstrate which one was more effective, because no statistical difference was observed between them (Table 3).

**Table 3.**
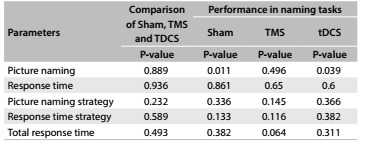
Mean performance in naming tasks from before to after stimulation (Wilcoxon test) and comparison of the techniques

TMS = transcranial magnetic stimulation; tDCS = transcranial direct current stimulation.

Descriptive analysis was conducted on the patients’ overall performance, from before to after stimulation. Four subjects demonstrated improvement in all tDCS parameters (patients 1, 9, 10 and 13) and subject #10 was randomly selected as the best responder. Since subject #12 did not show any improvement, this patient was considered to be the worst responder. The subject randomly selected as the control revealed intermediate results, with improvement in two parameters (Table 4).

**Table 4.**
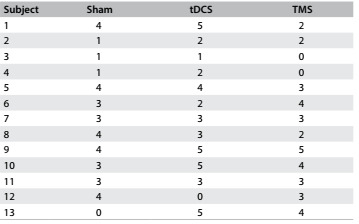
Improvement in parameters evaluated

tDCS = transcranial direct current stimulation; TMS = transcranial magnetic stimulation.

**Figure 1.**
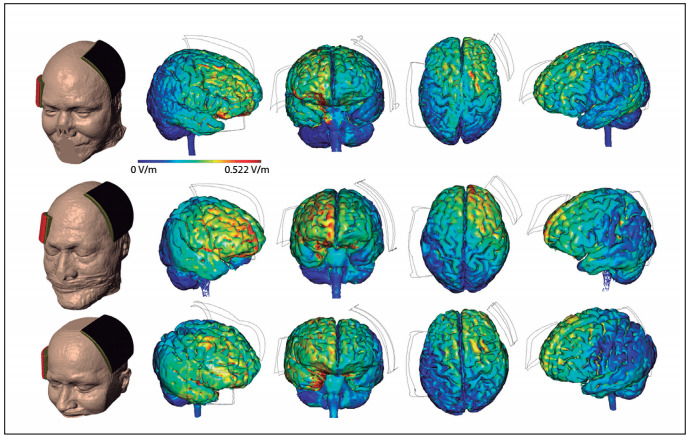
Peak intensities and distributions of cortical electric field (current density).

There were differences in the peak intensities and distributions of the cortical electric field (current density) between the subjects, as demonstrated in Figure 1.

The results shown in Figure 1 indicate that the subject with the best result (ranked first) showed less diffuse distribution in the right frontal lobe, with peaks in the area homologous to Broca’s area and in the orbital gyri. The subject with the worst result (ranked third) showed diffuse distribution over the right temporal and frontal lobes, with peaks in the right temporal lobe. The control subject (ranked second) had an intermediate result, showing diffuse distribution in the right frontal lobe, with peaks in the superior frontal gyrus.

## DISCUSSION

The number of studies using non-invasive brain stimulation in language areas is increasing and most of them have involved naming tasks for aphasic subjects in more than one session of stimulation.^1^^,^^7^^,^^19^^,^^20^ Investigations have indicated that better results were found when neuromodulation was combined with and speech therapy for language skills (especially regarding picture-naming accuracy and latency).^21^^,^^22^^,^^23^^,^^24^^,^^25^^,^^26^

The present study showed that there were statistically significant differences in picture-naming tasks after a single application of tDCS and after sham stimulation. The lack of detectable effect through TMS was possibly to be expected, given that high variability between subjects and weak effects regarding clinical outcomes from a single session have been shown.^9^^,^^27^^,^^28^^,^^29^ Moreover, in TMS, it is difficult to precisely locate the right place to apply the stimulus and small variations in coil positioning may generate stimulation in different regions.^30^ Hence, neuronavigation may help to determine the best location for the stimulus and improve the outcomes from TMS.

In neurorehabilitation, the challenge is magnified by inter-individual differences in injuries, along with slow and variable recovery rates even with effective treatment. Hamilton et al.^31^ suggested that the difficulty in knowing precisely which brain regions are affected by tDCS was one of the factors that limit expansion of use of this technique. One theory that could explain tDCS results is the current flow distribution. In this regard, computational models can predict the current flow density and may be an option for understanding the results or even for elaborating electrode setups.^17^^,^^18^ In the present study, computational models were used in post-hoc analysis and showed that the current flow distribution in the cortex differed among patients with different results. However, the number of patients in this study, which was calculated statistically, was too limited to prove this supposition.

## CONCLUSIONS

The first intervention did not show any statistically significant difference between tDCS, TMS and sham stimulation in any of the naming tasks and it was not possible to compare the techniques. Computational model procedures showed different current flow patterns among patients with different results from tDCS. This study supports the notion that the current flow may explain the different outcomes from the first intervention. However, the number of patients in this study was too limited to prove this supposition.

The limitations resulting from use of a single session of neuromodulation and the small number of subjects enrolled in the present study need to be taken into account. It would be advisable to conduct prospective controlled clinical trials with higher numbers of patients and multiple sessions of stimulation in order to establish a more precise approach and to compare tDCS and TMS.
